# Supplementary material for: Genetic drift acts strongly on within-host influenza virus populations during acute infection but does not act alone
Source: bioRxiv. 2025 Aug 30:2025.08.27.672713. Preprint. [Version 1] doi: 10.1101/2025.08.27.672713 (PMC12407903; doi:10.1101/2025.08.27.672713)
Supplement: Supplement 1 [file media-1.pdf]

Genetic drift acts strongly on within-host influenza virus populations during acute infection but does not act alone

Supplemental Material

Yike Teresa Shi[1], Michael A. Martin[2], Daniel Weissman[3], Katia Koelle[1,4,*]

1 Department of Biology, Emory University, Atlanta, GA, USA
2 Department of Pathology, Johns Hopkins School of Medicine, Baltimore, MD, USA
3 Department of Physics, Emory University, Atlanta, GA, USA
4 Emory Center of Excellence for Influenza Research and Response (CEIRR), Atlanta GA, USA

[*]katia.koelle@emory.edu

Supplemental Figures

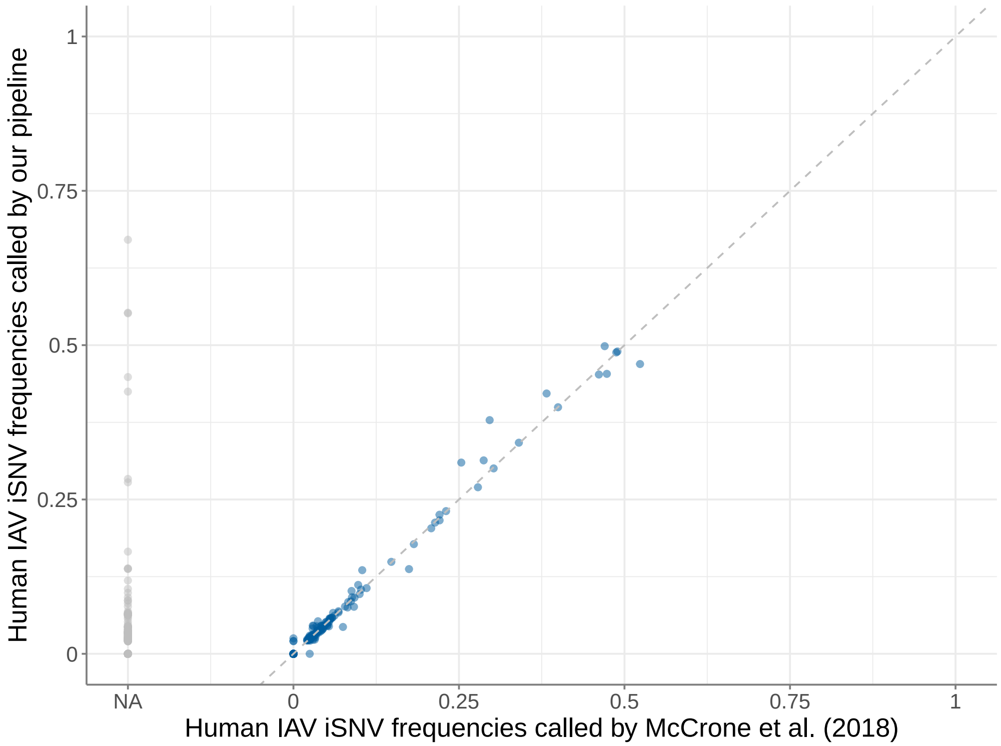

Figure S1. Comparison between iSNV frequencies called in the original analysis of McCrone et al. (2018) and those called by our pipeline (detailed in the Methods section).

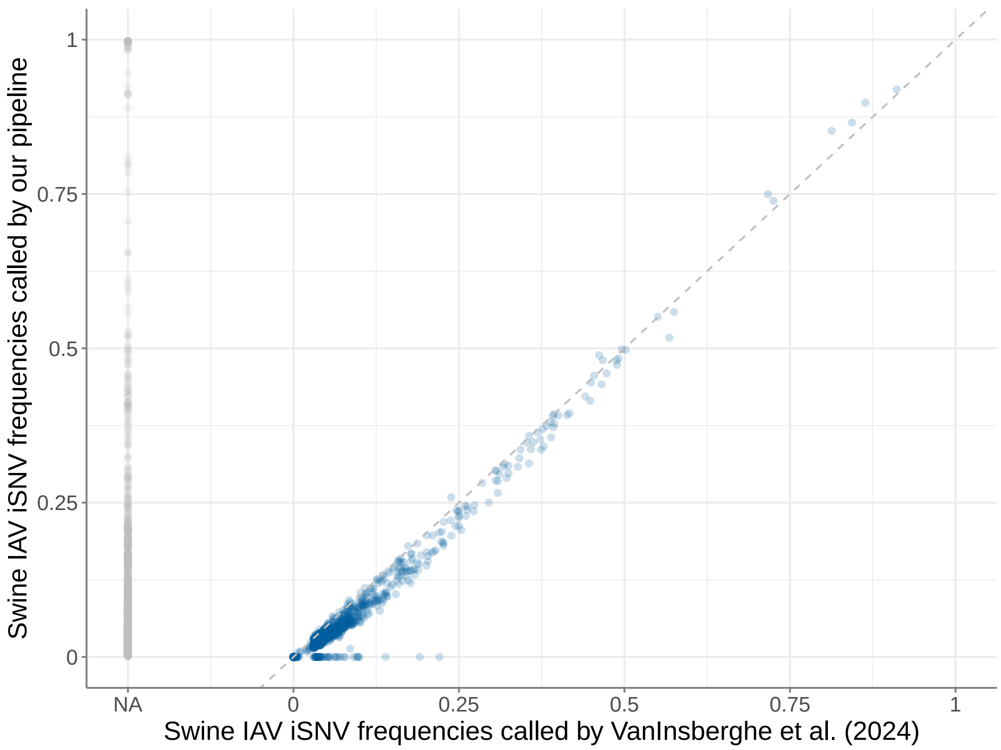

Figure S2. Comparison between iSNV frequencies called in the original analysis of VanInsberghe et al. (2024) and those called by our pipeline (detailed in the Methods section).

References

J. T. McCrone, R. J. Woods, E. T. Martin, R. E. Malosh, A. S. Monto, and A. S. Lauring. Stochastic processes constrain the within and between host evolution of influenza virus. *eLife*, 7:e35962, 2018.

D. VanInsberghe, D. S. McBride, J. DaSilva, T. J. Stark, M. S. Y. Lau, S. S. Shepard, J. R. Barnes, A. S. Bowman, A. C. Lowen, and K. Koelle. Genetic drift and purifying selection shape within-host influenza a virus populations during natural swine infections. *PLoS Pathog.*, 20(4):e1012131, Apr. 2024.
